# Design of Trials with Composite Endpoints with the R Package CompAREdesign

**DOI:** 10.1007/s12561-025-09488-3

**Published:** 2025-05-15

**Authors:** Jordi Cortés-Martínez, Marta Bofill-Roig, Guadalupe Gómez-Melis

**Affiliations:** 1 Research group in Biostatistics and Bioinformatics, GRBIO, Department of Statistics and Operations Research and Institute for Research and Innovation in Health (IRIS), Universitat Politècnica de Catalunya (BarcelonaTech), Jordi Girona 1-3, 08034 Barcelona, Spain; 2 Center for Medical Data Science, Medical University of Vienna, Spitalgasse 23, Vienna, 1090 Austria

**Keywords:** Composite endpoints, R package, Clinical trial, Survival analysis

## Abstract

Composite endpoints are frequently used as primary endpoints in clinical trials, especially those involving time-to-event data. Designing such trials poses significant challenges due to the frequent violation of the proportional hazards assumption when composite endpoints are used, even if the assumption holds true for individual components. Consequently, the conventional formulae for sample size calculation no longer apply. This paper introduces the R package CompAREdesign which incorporates novel methodologies developed by the authors to compute key trial design elements, including sample size and effect sizes, based on information of composite endpoint components. Additionally, the package offers features for designing trials with binary composite endpoints, and functions to simulate trials under a wide range of scenarios. While several R packages exist for analyzing trials with composite endpoints, CompAREdesign package is, to our knowledge, the first package specifically tailored to the design phase of such studies. CompAREdesign is available on CRAN.

## Introduction

Composite endpoints are defined as the occurrence of the set of events of interest in trials with binary endpoints and as the time from randomization to the first observed event among all events of interest in time-to-event trials. Composite endpoints are often chosen as primary efficacy endpoints to answer the main research question in confirmatory clinical trials. For instance, progression-free survival, the composite of the occurrence of death and clinical progression, is one of the most common primary endpoint in oncology clinical trials. Major adverse cardiovascular events (MACEs), the composite of cardiovascular death, myocardial infarction, stroke, and target vessel revascularization, is frequently used in cardiovascular research [13, 16, 36].

There is a large and established literature on designing and analyzing trials with multiple endpoints. The reader may refer to Rauch et al. [27] for methods for planning and evaluating clinical trials with primary composite endpoints, Sozu et al. [34] for sample size determination in trials with multiple endpoints, and Ristl et al. [29] for a revision of methods for the analysis of trials with multiple endpoints in small populations. In the specific context of composite endpoints, various approaches have been proposed. These include methods that weight the contributions of the composite’s individual components, as discussed by Ozga and Rauch [25] and Bakal et al. [2]. Additionally, matched-paired approaches, such as the win-ratio method, have been introduced by [12, 14, 26]. Methods for sample size calculation have also been developed, as detailed by Sugimoto et al. [35].

To our knowledge, only a few R packages in the R CRAN repository are exclusively focused on composite endpoints. Furthermore, those are more concentrated on the analysis rather than on the design. The **WR**, **WWR**, and **WINS** packages address the analysis of studies with prioritized composite endpoints through the Win Ratio measure. The **wcep** package provides Kaplan–Meier survival curves in the presence of weighted composite endpoints. The **Wcompo** implements inferential and graphic procedures for the semi-parametric proportional means regression of weighted composite endpoint of recurrent events and death. The **idem** package implements a procedure for comparing treatments based on the composite endpoint of a functional (unobserved) outcome and a time-to-event endpoint. On the other hand, there are a variety of packages for designing trials with multiple endpoints, but surprisingly, they do not consider composite endpoints. The **Mediana** library considers different multivariate distributions to calculate by simulation the sample size needed to analyze multiple events. **gMCP** and **gMCPLite** provide functions for the analysis of trials with multiple hypotheses using graph-based procedures. The **ADCT** package performs power and sample size calculations for adaptive designs with co-primary endpoints. Finally, **cats** allows us to simulate platform trials with co-primary binary endpoints.

The use of composite endpoints in clinical trial design is a well-established methodological approach, with several statistical frameworks available in the literature [5, 11, 18, 19, 25, 27]. Although these methodologies have been extensively studied, their practical implementation in trial design remains challenging.

This article introduces CompAREdesign [6], an R package that simplifies the application of these statistical methods in clinical trial planning, offering increased flexibility and reproducibility compared to existing web-based tools. CompAREdesign extends the functionalities of the CompARE web platform (https://compare-composite.github.io/compare) by allowing direct integration with other R packages, parameter customization, additional features, and simulation capabilities. This facilitates a more comprehensive and reproducible analysis workflow for clinical trial statisticians.

The main features of the **CompAREdesign** package are as follows: (1) to anticipate the relative efficiency of the design using a composite endpoint with respect to the design based on a single outcome as the primary endpoint; (2) to quantify the expected treatment effect of the intervention on the composite endpoint; and (3) to compute the sample size to detect that treatment effect. Although this paper mainly refers to trials with time-to-event endpoints and presents the R functions for that, the corresponding functions for trials with binary endpoints are implemented as well in **CompAREdesign**. We end this article with an overview of the functions of **CompAREdesign** for binary composite endpoints and a brief explanation of some functions to generate data for composite endpoints based on the information of the components.

The Methodology section outlines previously published statistical techniques, which are essential for providing context and helping users understand the theoretical framework behind the **CompAREdesign** package.

## Methodology

We begin by describing the methods implemented in the package. The following methods primarily correspond to the contributions made by the authors in their respective papers: [4, 5, 11, 18].

### Methodological Background

Consider an RCT comparing an experimental treatment ($i=1$) and a control arm ($i=0$). Suppose that $n^{(i)}$ patients are allocated to arm *i* and followed for a prespecified time $\tau$. Consider a composite endpoint ($\varepsilon_{\ast}$) consisting of two single events, $\varepsilon_1$ and $\varepsilon_2$, and assume that $\varepsilon_1$ is more relevant for the trial purposes. Denote by $T_1$ and $T_2$ the times to $\varepsilon_1$ and $\varepsilon_2$, respectively, and $T_{\ast}$ the time to the composite endpoint $\varepsilon_{\ast}$, that is, $T_{\ast}=\min \{T_1,T_2\}$ is the time to the first occurrence of the events $\varepsilon_1$ and $\varepsilon_2$. Furthermore, when needed, we denote by $T_k^{(i)}$ the time to $\varepsilon_k$ in the arm *i*.

### Distribution Functions for the Composite Endpoint

To derive the law for the composite endpoint, we have to distinguish between whether death (or any other fatal event) is included in $\varepsilon_1$ and $\varepsilon_2$. We will refer to case 1: when none of the events includes a fatal event that precludes from observing the other event (e.g., death); cases 2 and 3: when, respectively, either $\varepsilon_2$ or $\varepsilon_1$ includes a fatal event; and case 4: when both events include a fatal event. This distinction matters since, depending on the case, the cause-specific hazard rate function for $T_1$ and $T_2$ must be used instead of the corresponding marginal hazard rate functions. For simplicity, in this paper, we will focus on case 3 when the more relevant endpoint, $\varepsilon_1$, includes a fatal event. The reader is referred to [18] for a thorough explanation of the four cases.

To derive the law of $T_{\ast}^{(i)}$ , the joint distribution between $T^{(i)}_1$ and $T^{(i)}_2$ ($i=0,1$) has to be characterized. This is accomplished through a copula binding the marginal distributions of $T^{(i)}_1$ and $T^{(i)}_2$ through an association parameter that can be chosen between Spearman’s rank correlation coefficient $\rho$ and Kendall’s $\tau$. We assume that $\rho$ and $\tau$ are the same for both groups.

The marginal laws for $T^{(i)}_k$ ($i=0,1; k=1,2$) are chosen from the Weibull family of distributions. They depend on a shape parameter, which allows increasing, constant and decreasing hazard functions and a scale parameter that is specified in terms of the probabilities $p_1^{(0)}=p_1^{(0)}(\tau )$ and $p_2^{(0)}=p_2^{(0)}(\tau )$ of observing endpoints $T_1^{(0)}$ and $T_2^{(0)}$ in the control group.

Finally, we assume that treatment groups have proportional (cause-specific) hazard rates for each component and denote by $\mathrm{HR}_1$ and $\mathrm{HR}_2$ the respective (cause-specific) hazard ratios that have to be anticipated. Without loss of generality, we assume that both events $\mathcal{E}_1$ and $\mathcal{E}_2$ are harmful and the new treatment is expected to reduce the risk of both events, that is, $HR_k<1$, $k=1,2$ [11].

### Effect Size

In trials with survival endpoints, the efficacy of a treatment is routinely quantified using the hazard ratio based on the proportional hazards model. However, this proportionality is not usually met for time-to-event composite endpoints and different summaries, such as the geometric average hazard ratio, the average hazard ratio, the median ratio or the restricted mean survival time ratio could be a more convenient alternative [28, 39]. In what follows, we describe them.

**The geometric average hazard ratio**, $\mathrm{gAHR}$, is defined as the exponentiated mean of the logarithm of the hazard ratio, that is,

1 $$\begin{aligned} \mathrm{gAHR}=\exp \left\{\mathrm{E}(\log \mathrm{HR}_{\ast}(T)) \right\} \end{aligned},$$

where $\mathrm{HR}_{\ast}(t)$ is the all-cause hazard ratio of the composite endpoint $T^{\ast}$ and the expectation is taken for a given event-time distribution, which in this case is the average distribution of $T_{\ast}^{(0)}$ and $T_{\ast}^{(1)}.$ For a given maximum follow-up time $\tau$, the geometric average hazard ratio up to $\tau$ is defined as

2 $$\begin{aligned} \mathrm{gAHR}(\tau )= \exp \left\{ \frac{\int_0^{\tau } \log \big \{\frac{\lambda_{\ast}^{(1)}(t)}{\lambda_{\ast}^{(0)}(t)} \big \} f^{(a)}_{\ast}(t) dt}{p^{(a)}_{\ast}(\tau )} \right\} \end{aligned},$$

where $f^{(a)}_{\ast}(t)=(f_{\ast}^{(0)}(t)+f_{\ast}^{(1)}(t))/2$ is the average of the density functions of $T_{\ast}^{(0)}$ and $T_{\ast}^{(1)}$, $f^{(i)}_{\ast}(t)$ is the density function of $T^{(i)}_{\ast}$ ($i=0,1$), and $p^{(a)}_{\ast}(\tau )=(p^{(0)}_{\ast}(\tau )+p^{(1)}_{\ast}(\tau ))/2$ is the average probability of experiencing the event $\varepsilon_{\ast}$ over both groups by time $\tau$. Furthermore, the logrank test to compare the hazard rates of two groups under alternatives, where the hazard functions for the two groups are non-proportional, is approximately unit-variance normal, with a non-zero mean $\mu_{\ast} (\tau )$. This mean depends on the survival and censoring distributions in the two groups, and the proportion of subjects in each group. It is given by $\mu_{\ast}(\tau ) =\sqrt{n\pi (1-\pi ) p_{\ast}^{(a)}(\tau )} \log ( gAHR(\tau ) )$. This formula indicates that $\mu_{\ast} (\tau )$ depends on the geometric average hazard ratio without relying either on the proportionality of either the cause-specific hazard rates or the all-cause hazard rates, making $\mathrm{gAHR}(\tau )$ the natural effect measure when using the logrank test. For a comprehensive explanation of the relationship between the logrank test and the geometric average hazard ratio, the reader is referred to [11].

In contrast to $\mathrm{HR}_{\ast}(t)$ which is expected to change over time, $\mathrm{gAHR}(\tau )$ is independent of time, maintains its interpretability under non-proportional hazards, and should be used instead of the standard hazard ratio [31]. Furthermore, the geometric average hazard ratio and the all-cause hazard ratios take identical values under proportionality of the all-cause hazard rates.

**The average hazard ratio**, $\mathrm{AHR},$ introduced in [23], provides a summary statistic of the effect size that has an interpretation in the absence of proportionality among the hazard rates. The average hazard ratio up to time $\tau$ is defined as

3 $$\begin{aligned} \mathrm{AHR}(\tau )=\frac{\int_0^{\tau } (\lambda_{\ast}^{(1)}(t)/\lambda_{\ast}^{(a)}(t))f_{\ast}^{(a)}(t)dt}{\int_0^{\tau } (\lambda_{\ast}^{(0)}(t)/\lambda_{\ast}^{(a)}(t))f_{\ast}^{(a)}(t)dt} \end{aligned},$$

where $\lambda^{(a)}_{\ast}(t)=\lambda_{\ast}^{(0)}(t)+\lambda_{\ast}^{(1)}(t)$ is the overall hazard function among the two groups. The average hazard ratio up to time $\tau$ can be interpreted as an average of the hazard ratios at all death times, also under non-proportional hazards.

A previous work [11] showed, based on simulations, that the values of $\mathrm{AHR}$ and $\mathrm{gAHR}$ are very close, which means that $\mathrm{gAHR}$ could be interpreted as a measure of proportional hazards in the same way as $\mathrm{AHR}$. In addition, the $\mathrm{gAHR}$ has the advantage of a direct relationship with the sample size calculation, as explained in the next section.

**The median ratio**, $\mathrm{mR}_{\ast}$, corresponds to the ratio of the median times to $\varepsilon_{\ast}$ over both arms. Therefore, if $m_{\ast}^{(i)}=inf\{t: S_{\ast}^{(i)}(t)<0.5\}$ where $S_{\ast}^{(i)}(t)$ is the survival function of the composite endpoint in group *i* ($i=0,1$), then

4 $$\begin{aligned} \mathrm{mR}_{\ast}=\frac{m_{\ast}^{(1)}}{m_{\ast}^{(0)}}. \end{aligned}$$

The median ratio is another appropriate alternative to the $\mathrm{gAHR}(\tau )$ and the $\mathrm{AHR}(\tau )$, which coincides with those when the event rate is constant over time. Furthermore, the $\mathrm{mR}_{\ast}$ gives a measure of the time-to-event gain in one group relative to another, giving it greater interpretability than risk-based measures (e.g., $\mathrm{gAHR}(\tau )$ or $\mathrm{AHR}(\tau )$) [9].

**The restricted mean survival time ratio**, $\mathrm{RMSTR}_{\ast}(\tau )$, corresponds to the ratio of the restricted mean survival times to $\varepsilon_{\ast}$ over both groups up to time $\tau$. The restricted mean survival time (RMST) of $\varepsilon_{\ast}$ in arm *i* up to time $\tau$ is the area under the survival curve up to $\tau$, given by $\mathrm{RMST}_{\ast}^{(i)}(\tau )=\int_0^{\tau }S_{\ast}^{(i)}(t)dt$ [30]. The restricted mean survival time ratio is defined as follows:

5 $$\begin{aligned} \mathrm{RMSTR}_{\ast}(\tau )=\frac{RMST_{\ast}^{(1)}(\tau )}{RMST_{\ast}^{(0)}(\tau )}. \end{aligned}$$

Although the difference in restricted means as an alternative measure of treatment effect is often used, we advocate here for the ratio for analogy with all the other effect size measures.

### Sample Size

Sample size calculation to detect a hypothesized difference between treatments is a key point in the design of an RCT. In survival trials with composite endpoints, the sample size can be based on the geometric average hazard ratio, *gAHR*, in case the proportional hazards assumption can be assumed to hold for the components, but not for the composite endpoint. The required number of events, sample size, and power formulae are based on the non-centrality parameter $\mu_{\ast} (\tau )$ of the logrank test under the alternative hypothesis, which is a function of the *gAHR*.

Suppose we aim at testing the superiority of the new treatment $(i=1)$ against the control arm and that the logrank test statistic $Z_{\ast}$ is used for the null hypothesis of no effect on $T_{\ast}$. If using the geometric average hazard ratio as the treatment effect measure, the null hypothesis of no effect on $T_{\ast}$ will be rejected for a one-sided $\alpha$ significance level whenever $Z_{\ast}<-z_{\alpha },$ where $z_{\alpha }$ is the $\alpha$-quantile of the standard normal distribution. Note here that negative values of $Z_{\ast}$ favor the new treatment. Since $Z_{\ast}$ follows a normal distribution with mean $\mu_{\ast}(\tau )$ and variance 1, the power $1-\beta$ is such that $1-\beta =\mathrm{Prob}\{Z_{\ast}<-z_{\alpha }\}.$ Hence, the total sample size for both groups for a balanced design (equal sample size in both groups) is

6 $$\begin{aligned} n=\frac{4(z_{\alpha }+z_{\beta })^2}{p_{\ast}^{(a)}(\tau ) \left( \log (gAHR(\tau )\right)^2} \end{aligned}$$

and the expected number of composite endpoint events $e_{\ast}=n\cdot p_{\ast}^{(a)}(\tau )$ is given by

7 $$\begin{aligned} e_{\ast}=\frac{4(z_{\alpha }+z_{\beta })^2}{\left( \log (gAHR(\tau )\right)^2} \end{aligned}.$$

To obtain expression (7) from expression (2.4) (or vice versa), the same follow-up period ($\tau$) had to be assumed for all study participants, regardless of how recruitment was carried out. Observe that formula (7) corresponds to Schoenfeld’s formula [32] for the required number of events if the hazard ratio is replaced by the geometric average hazard ratio. However, the main difference is that while the Schoenfeld formula assumes that hazard rates are proportional when a composite endpoint is involved, all-cause hazard rates need not be.

To determine the number of events, and consequently the required sample size, Freedman’s formula [15]

8 $$\begin{aligned} e_F=\frac{(gAHR(\tau )+1)^2(z_{\alpha /2}+z_{\beta })^2}{(gAHR(\tau )-1)^2} \end{aligned}$$

can also be used. Both Freedman’s and Schoenfeld’s formulas are nearly correct when the number of events, $e_S$ and $e_F,$ is large and the groups are balanced. Since $\log x>2\frac{x-1}{x+1},$ for $x=gAHR(\tau )<1$ (and similarly for $x=gAHR(\tau ) > 1,$ as both formulas remain invariant against an exchange of the groups), Schoenfeld’s formula generally yields a lower required number of events. From a parsimonious perspective, Schoenfeld’s formula is preferable when minimizing the sample size is the main criterion. However, when the number of events is small, relying on asymptotic formulas becomes questionable, and computer simulations are recommended to ensure accuracy [1]. Whenever the groups are not balanced, the variance of the log-hazard ratio estimate increases, affecting the required number of events and the Freedman formula that accounts for the allocation ratio is more appropriate [21].

### Endpoint Selection

When designing a trial with multiple endpoints, one might wonder which endpoint to choose from a point of view of the statistical efficiency of the trial.

Especially when composite endpoints are considered, adding more endpoints to the primary composite endpoint can dilute the effect of some of the most relevant endpoints. The asymptotic relative efficiency (ARE) was proposed as a measure to evaluate the statistical efficiency gain of the composite endpoint versus one of its components. Specifically, the ARE for survival composite endpoints compares the efficiency of using the logrank test $Z_{\ast}$ based on the composite endpoint $\varepsilon_{\ast}$ versus the logrank test *Z* based on the relevant endpoint $\varepsilon_1.$ In what follows, we sketch the main idea of the method, but for further details, we refer to the article of [18].

Given that both tests *Z* and $Z_{\ast}$ are asymptotically N(0,1) under $H_0$: no effect on $T_1$ and $H_0^{\ast}$: no effect on $T_{\ast}$ and are asymptotically normal with variance 1 under a sequence of contiguous alternatives to the null hypothesis, the ARE is given by the square of the ratio of their non-centrality parameters $\mu$ and $\mu_{\ast},$ respectively, and admits the following expression:

9 $$\begin{aligned} \mathrm{ARE}(Z_{\ast}, Z)=\left( \frac{ \mu _*}{\mu }\right) ^2= \frac{\left( \int _0^{1} \log \big \{\lambda _{*}^{(1)}(t)/\lambda _{*}^{(0)}(t) \big \} f_*^{(0)}(t) \mathrm{d}t\right) ^2 }{ (\log \mathrm{HR}_{1})^2 (\int _0^{1} f_*^{(0)}(t) \mathrm{d}t) ( \int _0^{1} f^{(0)}_1(t) \mathrm{d}t) } \end{aligned}$$

where $f_1^{(0)}(t)$ and $f_{\ast}^{(0)}(t)$ correspond to the densities, under the control group, of $T_1$ and $T_{\ast},$ respectively, $\lambda_{\ast}^{(0)}(t)$ and $\lambda_{\ast}^{(1)}(t)$ correspond to the hazard functions of $T_{\ast}$ under the control and experimental groups, respectively, and $\mathrm{HR}_{1}$ stands for the constant hazard ratio for endpoint $T_1.$ The ARE measure can be roughly interpreted as the ratio of the required sample sizes using $\varepsilon_1$ versus $\varepsilon_{\ast}$ to attain the same power for a given significance level. This measure therefore yields the following criterion: whenever ARE >1, choose $\varepsilon_{\ast}$ as the primary endpoint to guide the study; otherwise, use $\varepsilon_1.$

## CompAREdesign R Package

In this section, we provide a general description of the package. We start by explaining the installation and dependencies and continue describing the functions and arguments for time-to-event functions. We postpone to a subsequent section the description of the functions for composite binary endpoints. **CompAREdesign** provides either numerical or graphical outputs for all methods described in the methodological section for the design of trials with composite endpoints. Further details on the usage of the functions can be found in the corresponding R package manual.

### Installation and Dependencies

The **CompAREdesign** package is available on CRAN at https://CRAN.R-project.org/package=CompAREdesign and can be installed and loaded by running the R commands:
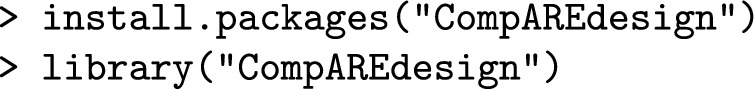


The package depends on the **copula** package [20], which implements joint distributions binned by copulas; the packages **ggplot2** [38] and **ggpubr** [24] needed for the graphical tools; and the packages **rootSolve** [33] and **numDeriv** [17] required to numerically compute some integrals.

### Explanation of Functions

**CompAREdesign** consists of 13 functions. Six of them refer to time-to-event composite endpoints, and seven refer to the binary composite endpoint. The functions whose name ends with tte are those for the time-to-event case, cbe for the binary case, and two extra functions named lower_corr and upper_corr concern the bounds of the correlation between binary endpoints. All these functions are implemented in the shiny web tool **CompARE** allowing an interactive way of using them. Table 1 gives a high-level description of these functions and relates them to the capabilities of the app.

**Table 1:** R functions included in the **CompAREdesign** package along with the corresponding description and the CompARE webtool’s tab where the function is used

R function	Description	CompARE web tool tab
* Functions for the composite of time-to-event endpoints*		
surv_tte	Computes the survival function for the composite endpoint and both components	Summary
effectsize_tte	Computes the treatment effect for the composite endpoint	Effect size
samplesize_tte	Computes the sample size for the composite endpoint	Sample size
ARE_tte	Computes the ARE method for time-to-event endpoints	Endpoint selection
plot_tte	Returns four plots related to previous features	All tabs
simula_tte	Simulates time-to-event data for the composite and its components	(Not implemented)
*Functions for the composite of binary endpoints*		
prob_cbe	Computes the probability of observing the composite	Summary
lower_corr	Computes the lower limit for Pearson’s correlation	Association measures
upper_corr	Computes the upper limit for Pearson’s correlation	Association measures
effectsize_cbe	Computes the expected treatment effect for the composite endpoint	Effect size
samplesize_cbe	Computes the needed sample size for the composite endpoint	Sample size
ARE_cbe	Computes the ARE method for composite endpoint	Endpoint binary endpoints
simula_cbe	Simulates binary data for the composite and its components	(Not implemented)

In what follows, we describe the time-to-event functions included in the package:

- **surv**_**tte** draws, in the same graphical window, for each treatment arm, the survival functions of each component as well as of the composite endpoint.
- **effectsize**_**tte** provides the anticipated treatment effect for the composite endpoint in terms of the following measures: the geometric average hazard ratio $gAHR_{\ast}(\tau$), the average hazard ratio $AHR_{\ast}(\tau$), the median ratio $mR_{\ast}$, and the restricted mean survival time ratio $RMSTR_{\ast}(\tau$). In addition, for each treatment arm, this function returns: the $RMST_{\ast}^{(i)}$, the $m_{\ast}^{(i)}$ , and the probability of observing the event in each group, $p_{\ast}^{(i)}$ ($i=0,1$).
- **samplesize**_**tte** returns the needed sample size for the three following designs, which depend on which primary endpoint is used: (1) sample size for a design based on the relevant component of the composite endpoint $\varepsilon_1$; (2) sample size for a design based on the second component $\varepsilon_2$; and (3) sample size for a design based on the composite endpoint $\varepsilon_{\ast}$.
- **ARE**_**tte** provides the Asymptotic Relatively Efficiency (ARE) value for comparing the efficiency of using a design with the composite endpoint as the primary endpoint versus a design with the first component as the primary endpoint. An ARE value larger than one indicates a benefit, in terms of power efficiency, when using a composite endpoint, and otherwise, the relevant endpoint is the preferred one.
- **plot**_**tte** is a summary function that draws the following four plots: (1) the survival function of the composite endpoint for each treatment arm; (2) the expected hazard ratio over the follow-up time; (3) the ARE value with respect to the correlation between components; and (4) the required sample size as a function of the correlation.
- **simula**_**tte** simulates two-arm trials with composite endpoints.

### Explanation of the Parameters

Most functions in the **CompAREdesign** package use common arguments. All these arguments are briefly described in Table 2. Note that in some cases, the same arguments are used for time-to-event and binary functions.

**Table 2:** Arguments of the functions for binary (*B*) and time-to-event (*T*) endpoints and their description

Argument	Description	B	T
p0_e1, p0_e2	Probability of occurrence of $\varepsilon_1$ and $\varepsilon_2$ in the control arm	X	X
eff_e1, eff_e2	Anticipated effect for the composite component $\varepsilon_1$ and $\varepsilon_2$	X	
effm_e1, effm_e2, effm_ce	Effect measure used for the event $\varepsilon_1$, $\varepsilon_2$ and $\varepsilon_{\ast}$	X	
HR_e1, HR_e2	Expected cause-specific hazard ratio for the $\varepsilon_1$ and $\varepsilon_2$		X
beta_e1, beta_e2	Shape parameter of a Weibull distribution for the $\varepsilon_1$, $\varepsilon_2$ in the control arm		X
rho	Pearson, Spearman or Kendall correlation between $\varepsilon_1$ and $\varepsilon_2$	X	X
rho_type	Type of correlation (Character)	X	
case	1 to 4 depending on whether death is included in $\varepsilon_1$ or $\varepsilon_2$ or both or neither	X	
copula	Type of copula to build the joint distribution	X	
alpha	Probability of Type I error	X	X
beta	Probability of Type II error	X	
power	Power to detect a real treatment effect on composite endpoint		X
ss_formula	Formula for the sample size calculation on the single components		X
unpooled	Variance estimate used for the treatment effect	X	
followup_time	Time of follow-up ($\tau$)		X
subdivisions	Number of points to perform numerical calculations		X
plot_print	Indicates if the plot should be displayed		X
plot_save	Indicates if the plot should be saved		X
sample_size	Desired sample size for each arm when simulating data		X

**p0**_**e1** and **p0**_**e2** represent the probabilities of observing the event in the reference arm during the follow-up period. It must be kept in mind that, for a single participant, p0_e1 is the probability of observing the $\varepsilon_1$ even if $\varepsilon_2$ had previously been observed and vice versa. Those probabilities could be easily obtained from the literature, for example, using the proportion of observed events at the end of the study in trials where the pertinent component is used as the primary endpoint.

**HR**_**e1** and **HR**_**e2** are the anticipated treatment effects for the composite components $\varepsilon_1$ and $\varepsilon_2$ in terms of the cause-specific hazard ratios. They could also be found in previously published trial results with $\varepsilon_1$ or $\varepsilon_2$ as primary endpoints.

The arguments **beta**_**1** ($\beta_1$) and **beta**_**2** ($\beta_2$) are the shape parameters of the marginal Weibull distribution for each component. They can be anticipated taking into account that values below unity imply a decreasing risk of that event occurring over time and vice versa, while a $\beta_j=1$, $j=1,2$ indicates a constant risk. Our recommendation is as follows:

- If the risk of suffering the event decreases throughout the follow-up period (e.g., an infection after surgical intervention), then assign values $\beta_j=0.5, j=0,1.$
- If the risk of suffering the event remains constant during the follow-up period (e.g., death after a non-invasive intervention in mild patients), then choose $\beta_j=1.$
- If the risk of suffering the event increases during the follow-up period (e.g., contracting an infectious disease at the start of a pandemic), then select $\beta_j=2.$

The ARE method is based on a copula binding the distribution of the times to $\varepsilon_1$ and $\varepsilon_2$ through a measure of association, for example, Spearman’s rank correlation coefficient (**rho**) between these times. As it can be hard to anticipate a measure of association, the package provides both the sample size and the ARE for different positive correlation values (we assume that the association between components cannot be negative). Time-to-event functions implement another association measure, Kendall’s $\tau,$ that could be used instead of Spearman’s $\rho$ by means of the argument **rho**_**type**. A note of caution is that Spearman’s $\rho$ cannot be obtained from the data in the presence of competing risk, i.e., when $\varepsilon_1$ and/or $\varepsilon_2$ is death.

The **case** parameter indicates in which of the two components that make up the composite event, a fatal event is present. This is important when addressing the scenario of competing risks:

- If case = 1, then none of the events of interest includes a fatal event that precludes from observing other events (such as death)
- If case = 2, then $\varepsilon_2$ is a fatal event
- If case = 3, then $\varepsilon_1$ is a fatal event
- If case = 4, then both components are fatal events. An example of this is cause-specific mortality, such as the composite includes deaths by different causes (e.g., death from heart disease, death from cancer, death from other causes, etc.)

The **copula** argument indicates which type of copula is used to obtain the joint distribution. Currently, the Archimedean copulas of Frank (default), Gumbel, and Clayton are implemented. The former provides the same weight to all the events over time, while the Gumbel and Clayton copulas provide more weight at the start and end of the follow-up, respectively [37].

**alpha** and **power** are relevant parameters for the sample size calculation. **alpha** ($\alpha$) represents the probability of Type I error, that is, the probability of finding a statistically significant treatment effect when it truly does not exist. The **power** ($1-\beta$) is the desired probability of detecting a treatment effect when it truly exists. The parameter **ss**_**formula** gives the user two options to calculate the needed number of events: Schoenfeld’s or Freedman’s formula.

**followup**_**time** ($\tau$) argument represents the length of the follow-up (measured in any time unit). It facilitates the interpretation of some graphics, since the range of the x-axis is fitted to this follow-up period. Moreover, time-dependent effect measures, such as $mR_{\ast}$ or the $RMST_{\ast},$ are calculated taking into account this value. By default, a unitary time is assumed (e.g., 1 year).

The purpose of the parameter **subdivisions** is to set in how many points some functions (e.g., $\mathrm{HR}^{\ast}$) are evaluated to be plotted. The higher the value, the more accuracy at a higher computational cost.

Finally, **plot**_**print** and **plot**_**save** indicate if the plots returned by some functions should be displayed and saved (for further customization), respectively.

## Illustration of CompAREdesign for Time-to-Event Endpoints

This section presents the main features of the package through a cardiovascular trial. This illustration exemplifies those aspects of the design of an RCT that can benefit from the capabilities of the functions implemented in this package.

### Planning a Cardiovascular Trial in Patients Based on SCORED Trial

We present a case study to illustrate how to use CompAREdesign to plan a new trial based on results of a previous trial, namely the SCORED trial [3]. The SCORED trial [3] compared sotagliflozin versus placebo in patients with diabetes and chronic kidney disease. The trial’s primary endpoint was a composite (CE, $\varepsilon_{\ast}$) comprising cardiovascular deaths (DCC $\varepsilon_1$) and hospitalizations or urgent visits for heart failure (HUHF, $\varepsilon_2$).

For our case study, let us assume that we aim to conduct a new RCT comparing two groups using the same endpoints as in the SCORED trial, so we will use information from the SCORED trial to decide on the assumed values.

Some of the required argument values for the functions are extracted from the published article, which reports i) the cause-specific HRs for each component: 0.90 for DCC ($\varepsilon_1$) and 0.67 for HUHF ($\varepsilon_2$); and ii) the probabilities of observing both components (DCC and HUHF) in the control arm: 0.024 and 0.051, respectively. However, certain assumptions must be made because of the lack of detailed information on the individual components. For example, we assume that the risk of DCC remains constant ($\beta_1=1$) over time, while the risk of HUHF increases ($\beta_2=2$). Furthermore, we could anticipate a moderate correlation ($\rho =0.3$) between both components. Before using the package, the first step is to specify all the necessary arguments for designing our RCT:
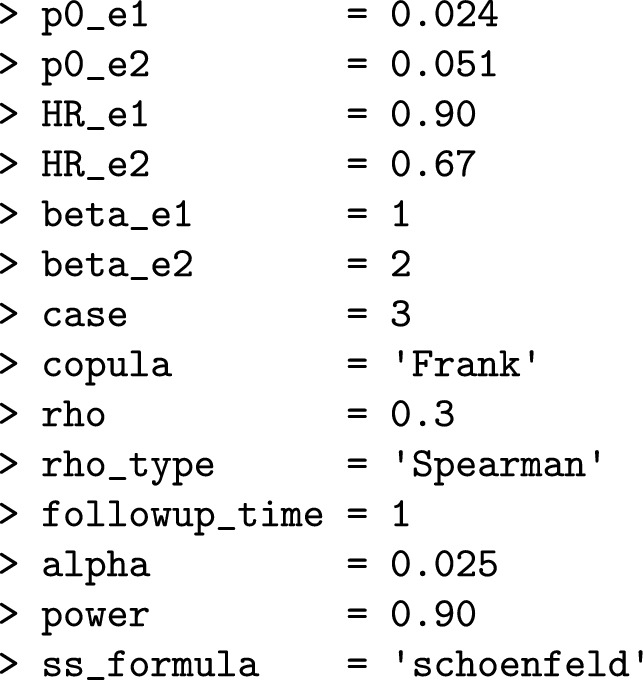


### General Overview and Characterization of the Law of $T_{\ast}$

**Fig. 1:**
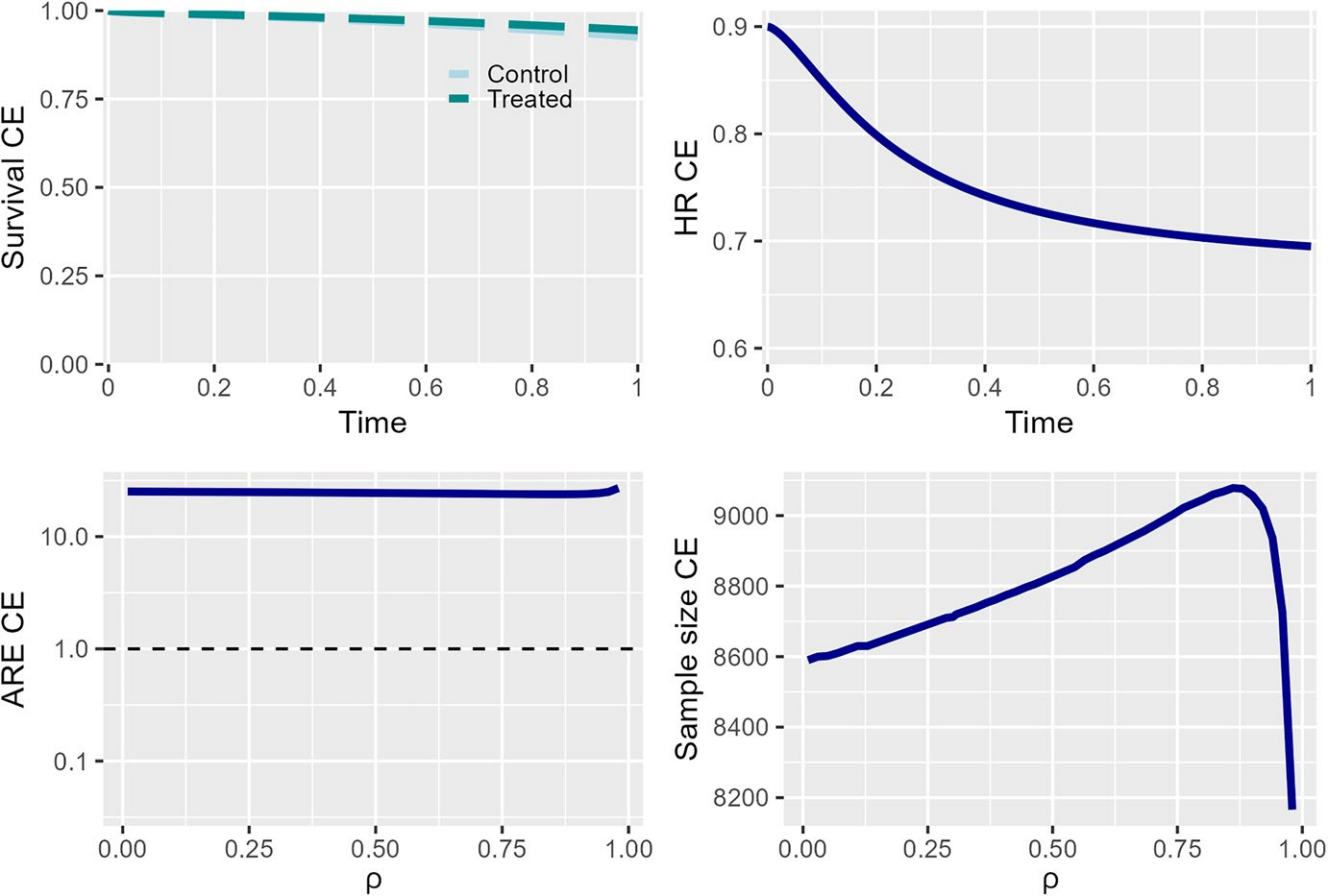
Top left: survival curves of the composite endpoint for both treatment arms. Top right: anticipated $\mathrm{HR}_{\ast}(t)$ over time. Bottom left: asymptotic relative efficiency as a function of the correlation between components. Bottom-right: needed sample size depending on the correlation between components

The plot_tte function is designed as an all-in-one function that displays all relevant plots for decision-making in an RCT. Figure 1 illustrates the key graphics used to assess the behavior of the CE in the trial design.
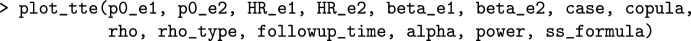

The top two plots in Fig. 1 display the survival and hazard ratio functions of the composite endpoint over time. The survival curves (top-left) reveal a significant amount of censored data in both treatment arms due to the low proportion of expected events by the end of the study (0.024 and 0.051 for $\varepsilon_1$ and $\varepsilon_2,$ respectively). To obtain survival curves for each component, the surv_tte function can be called using the same input parameters as plot_tte. The follow-up period can be specified to plot the survival curves at the relevant scale for a better interpretation. For example, we can set the followup_time argument to indicate a planned 2-year follow-up for the participants:
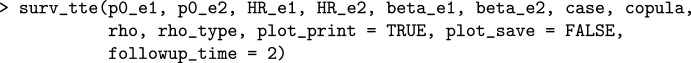


On the top-right of Fig. 1, the hazard ratio of the composite endpoint does not remain constant over time. Specifically, $\mathrm{HR}_{\ast}(t)$ decreases from $HR_{\ast}(0)\approx 0.90$ to $HR_{\ast}(0.5)\approx 0.70$ during the follow-up period. This plot is helpful for trial planning, as it allows for a visual assessment of whether the proportional hazards assumption holds in a specific setting.

As the association between the composite components, in the planning phase of a trial, is commonly unknown, our package provides the ARE and the sample size as a function of the selected association measure.

The bottom-left plot of Fig. 1 allows the user to assess the potential impact of correlation on the ARE value. In this particular example, the design using the composite endpoint as the primary endpoint is more efficient than the design considering DCC ($\varepsilon_1$), as the ARE remains approximately 24 regardless of the Spearman’s correlation between components. Therefore, in this context, the value of $\rho$ should not guide the decision to use the CE.

On the other hand, on the bottom-right plot, we can observe the sample size of the composite endpoint in terms of the correlation $\rho.$ For sensible values of the Spearman’s correlation ($\rho <0.90$), the sample size takes values between 8600 and 9200, slightly lower than the 10,500 patients planned for the SCORED trial.

### Effect Size Calculation by Means of effectsize_tte

As explained in the methodology section, the hazard ratio is a common effect measure in survival trials, but it relies on the proportional hazards’ assumption. Alternative effect measures have been proposed that may be more appropriate when the hazard ratio deviates from this assumption. The function effectsize_tte allows the user to anticipate the expected treatment effect for the CE based on the information from the individual components. The function returns the treatment effect using different measures, along with a plot of $\mathrm{HR}_{\ast}(t)$ over time. To compute the anticipated effect for the hypothetical trial based on the SCORED trial, we can use the following:
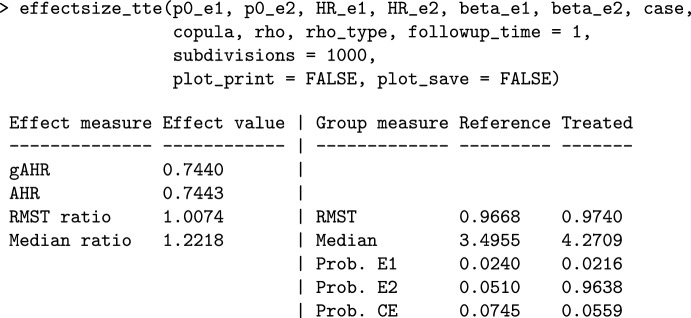


The output gives two summary measures of the $\mathrm{HR}_{\ast}(t)$ over time: the geometric average hazard ratio $gAHR_{\ast}(\tau )$ (see (2)) and the average hazard ratio $AHR_{\ast}(\tau )$ (see (3)) with quite similar values (both close to 0.74). When *HR*(*t*) is not constant, the $gAHR_{\ast}(\tau )$ and the $AHR_{\ast}(\tau )$ should be cautiously interpreted, since they do not reflect an overall effect applicable to the entire follow-up time. The function also reports other effect measures: the ratio of the restricted mean survival times up to the end of follow-up ($\tau$) $RMSTR_{\ast}(\tau )$ (see (5)), and the median ratio $mR_{\ast}(\tau )$ (Eq. (4)). The latter take a value of approximately 1.22, indicating that the intervention provides a 22% gain in the median survival time to event $\varepsilon_{\ast}$. Finally, some measures for each treatment arm are provided, such as the *RMST*, the median, and the probability of observing the events. The output also includes the plot of the $\mathrm{HR}_{\ast}(t)$ (not shown here).

The medians for the composite endpoint in each arm can always be calculated using the marginal Weibull distributions. However, results should be taken with caution when some of these medians go beyond the follow-up time ($\tau$).

### Sample Size Calculation by Means of samplesize_tte

We implemented the sample size calculation for survival trials with composite endpoints in the function samplesize_tte. This function allows the user to compute the sample size needed to have $1-\beta$ power to detect the effect of treatment at the significance level $\alpha$. The function samplesize_tte returns the total sample size (assuming equal-sized arms) that would be required if the trial is designed to detect an effect on the composite endpoint. For comparison, the needed sample sizes for trials using endpoint 1 ($\varepsilon_1$) or endpoint 2 ($\varepsilon_1$) as the primary endpoint are also provided.

We show the usage of the function using the SCORED trial as an example. We set the power and significance level equal to 0.90 and 0.025, respectively. The usage of samplesize_tte is as follows:
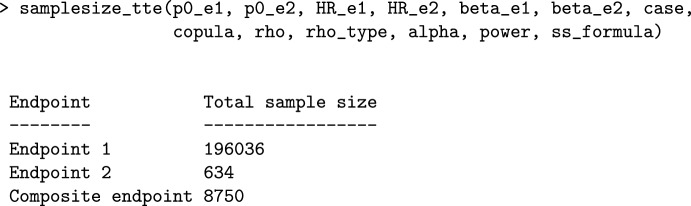


Note that the design that requires a smaller sample size is the one that considers HUHF ($\varepsilon_2$) as the primary endpoint. This is because the effect for endpoint 2 is larger than the one for endpoint 1, and therefore, it requires fewer participants to have 0.90 probability to detect the treatment effect. Furthermore, the number of expected observed events is also higher for HUHF (0.051 versus 0.024), which also makes the sample size for the design that only considers endpoint 1 ($\varepsilon_1,$ DDC) larger than that using endpoint 2. The use of the CE as the primary endpoint leads to a notably reduction of the sample size in comparison to using the design that considers $\varepsilon_1$ as the only relevant event.

**Fig. 2:**
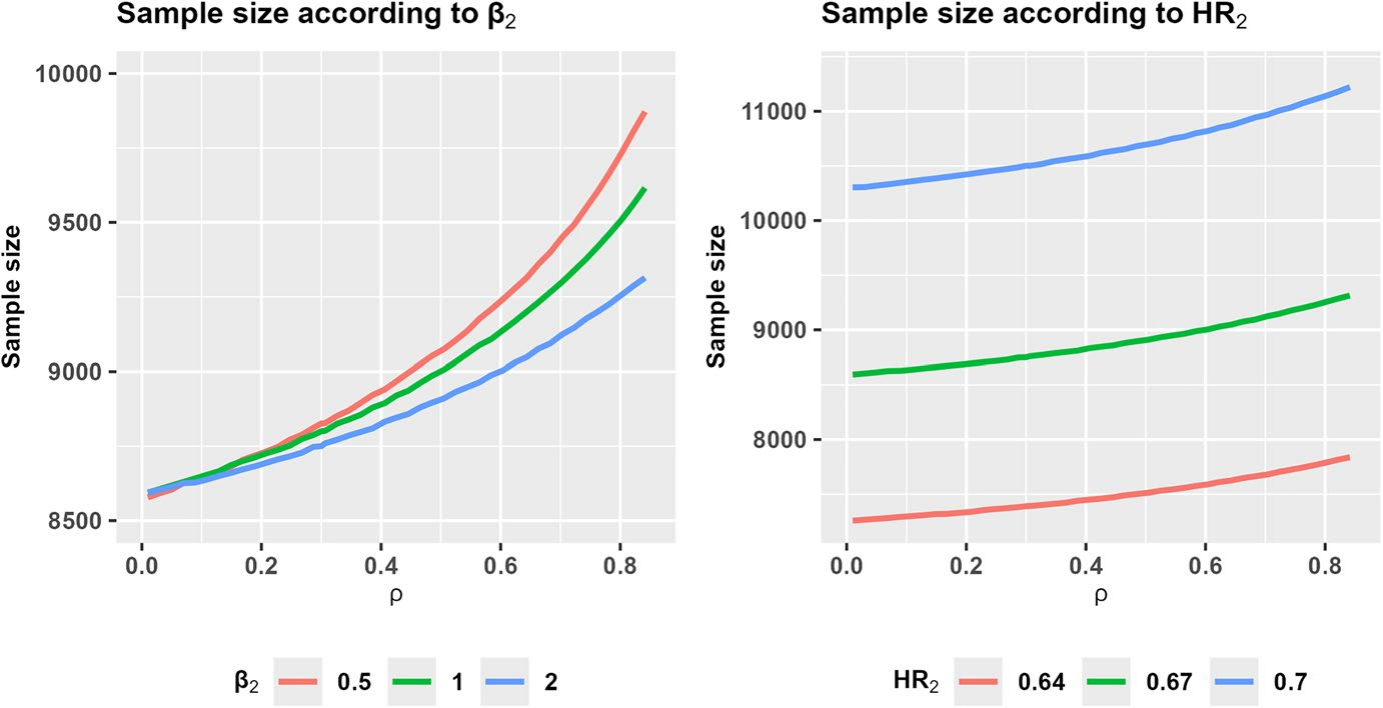
Sample size under several scenarios. On the left, sample size stratified according to the shape parameter $\beta_2$ of $\varepsilon_2$: decreasing risk over time (red, $\beta_2=0.5$); constant risk (green, $\beta_2=1$); and increasing risk (blue, $\beta_2=2$). On the right, sample size stratified according to the hazard ratio $\mathrm{HR}_2$ of $\varepsilon_2$: large treatment effect (red, $\mathrm{HR}_2=0.64$); moderate treatment effect (green, $\mathrm{HR}_2=0.67$); low treatment effect (blue, $\mathrm{HR}_2=0.70$). In both plots, values are represented over different values of Spearman’s rank correlation coefficient $\rho$ (Color figure online)

The sample size calculation is generally sensitive to the values of the parameters on which it relies. The choice of certain input arguments in the samplesize_tte function might significantly modify the required number of trial participants. CompAREdesign can be useful to evaluate the robustness or sensitivity of the sample size calculations according to the assumptions and parameter inputs. As an example, Fig. 2 shows the sample size calculation according to the Weibull shape parameter ($\beta_2$) and the treatment effect ($HR_2$) for the second component. If there is low or no association between both components, the choice of $\beta_2$ barely impacts the total sample size, which ranges between 8600 and 8700. The discrepancies are more extensive as the correlation increases. For example, for Spearman’s correlation equal to 0.80, the total size could range from 9250 if the DP risk increases over time to more than 9700 if the hazard decreases. Modifying the treatment effect is more critical; the right graph shows that considering a slight increase in the HR from 0.67 to 0.70 implies an increase in the total sample size from 8750 to 10,500. These plots were obtained by setting the parameter plot_save and later, manipulating the returned ggplot class object obtained through the **ggplot2** package [38].

### Endpoint Selection by Means of ARE_tte

The ARE gives a criterion to decide whether to use a composite endpoint ($\varepsilon_{\ast}$) or to use its more relevant component ($\varepsilon_1$) as the primary endpoint of the trial. Returning to the example based on the SCORED trial, the decision translates into whether to consider death by Cardiovascular disease (i.e., DCC) or a composite endpoint as the primary endpoint.

The ARE method is implemented in the ARE_tte function. To compare the two previously mentioned designs, the user can use the following code:
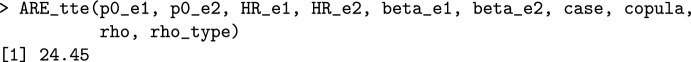


Note that a value of $\mathrm{ARE}=24.45>1$ implies that is more efficient considering the composite endpoint than the primary endpoint (DDC). This higher efficiency might result in a smaller required sample size if using PFS instead of OS to attain the same power for a given significance level.

## CompAREdesign for Binary Composite Endpoints

**CompAREdesign** also includes functions for the design of trials with composite binary endpoints. In this case, composite endpoints are defined as the occurrence of either one of the components.

Analogous to the time-to-event case, the distribution function for composite binary endpoints relies on the marginal event rates and effect sizes of the composite components, and the correlation between them [5]. As it is well known, when assessing the efficacy of an intervention against a control treatment based on a binary endpoint, we could use the difference in proportions (or risk difference), the relative risk (or risk ratio), and the odds ratio. The same applies for the composite binary endpoints. In **CompAREdesign**, we have implemented the effect size and sample size calculation for trials with composite binary endpoints based on the anticipated values of the composite components and their correlation according to risk difference, relative risk, and odds ratio effect measures. An overview of the functions implemented for binary endpoints is found in Table 1. In what follows, we briefly describe the two main functions, effect_cbe and samplesize_cbe, for the effect size and sample size computation, respectively.

The function effect_cbe can be used to compute the effect size of the composite binary endpoint by means of
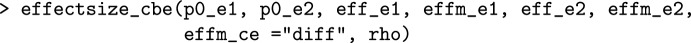


where p0_e1 and p0_e2 denote the probabilities of $\varepsilon_1$ and $\varepsilon_2$ in the control group, respectively; eff_e1 and eff_e2 are the anticipated effects for the events $\varepsilon_1$ and $\varepsilon_2$, respectively; and rho is Pearson’s correlation between $\varepsilon_1$ and $\varepsilon_2$. The effects for $\varepsilon_1$ and $\varepsilon_2$ can be anticipated in eff_e1 and eff_e2 by means of the difference of proportions, risk ratio, and odds ratio. The arguments effm_e1 and effm_e2 can be used to specify the preferred effect measure. Additionally, using the argument effm_ce, we specify the effect measure we are interested in for the composite endpoint.

The function samplesize_cbe can be called by
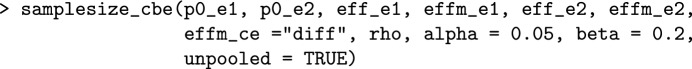


where p0_e1, p0_e2, eff_e1, eff_e2, and rho effm_e1, effm_e2, and effm_ce are the parameters explained above; and where alpha and beta are the type I and type II errors, respectively; and unpooled denotes the variance estimate used for the sample size calculation (TRUE for unpooled variance estimate, and FALSE for pooled variance estimate).

Other functions included in the R package are prob_cbe to calculate the probability of the composite endpoint, and ARE_cbe to compute the ARE method for binary endpoint, as proposed in the article of [4].

## Simulation with **CompAREdesign**

As an additional feature, the package **CompAREdesign** includes functions to generate data from both the components and the composite endpoint. Using the same input parameters previously seen, the simula_tte function generates samples_size times for each treatment arm and the corresponding censoring indicator variables. We assume non-informative administrative right censoring data, thus, all events not occurring before or at time $\tau$ (followup_time) are censored at $\tau$.

Simulations are in general a key tool in the design of RCTs to test the robustness of methods with respect to deviations of the assumptions, sensitivity of sample size calculations regarding the parameter values’ assumptions, and compare several methods (e.g., a trialist might wish to compare a non-parametric, a semi-parametric and a parametric method under certain conditions) and/or the efficiency of different trial designs.

The function returns a data frame with seven columns. The first 6 represent the three times (to event or to censoring) and the three censoring indicators (*status*) for $\varepsilon_1$, $\varepsilon_2$, and $\varepsilon_{\ast}$, respectively. The last column represents the treatment arm. The following example shows simulated data with similar characteristics as those from the SCORED trial.
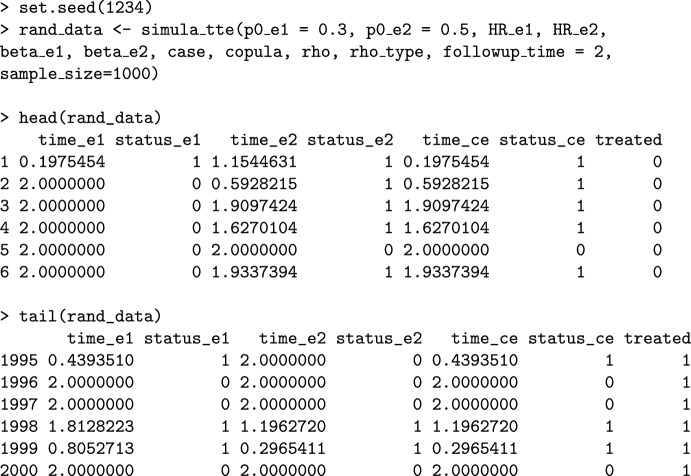


In the same way, simula_cbe simulates two-arm trials with binary composite endpoints.

## Conclusions

While several R packages exist for analyzing trials with composite endpoints, to our knowledge, the **CompAREdesign** package is the first specifically implemented to address the design of RCTs with composite endpoints. The design and analysis of trials with composite endpoints might be especially challenging, as it involves multiple assumptions about the components. For instance, it depends on the functional form of the survival distribution (e.g., the shape parameters of the marginal Weibull distributions) as well as on parameter values, such as the expected effect sizes, that is, the hazard ratios for each composite component. Additionally, the joint distribution between both component endpoints—including the marginal law and the association between both endpoints—must be anticipated. Since anticipation of this long list of arguments might be arduous, **CompAREdesign** can be a great help in the design of the trial.

The CompARE shiny web application and the R package serve complementary roles. The web platform offers an intuitive and user-friendly interface, making it particularly useful for initial exploration of how treatment effects evolve over time and for estimating the required sample size. In contrast, the CompAREdesign R package is designed for users who need a more rigorous statistical analysis, providing greater flexibility, customization, and integration with other statistical tools within the R ecosystem.

The **CompAREdesign** package may be of particular interest in cases where the precise value of these parameters is unknown. The sensitivity and robustness of results due to variations in initial values and assumptions can be assessed by means of this package. For instance, by means of the function samplesize_tte, one could compare several scenarios depending on the shape parameters of the Weibull distribution ($\beta_j$) or the expected effect size in either component ($\mathrm{HR}_j$) as seen in Fig. 2. In addition, **CompAREdesign** includes several association measures and copula functions for addressing different dependency structures between the time to each of the composite components. Although the choice of the copula is not straightforward, preliminary simulation results [10] show a small impact of the choice of the copula on the corresponding percentiles for the composite endpoint, and consequently on the decisions one might have to make.

**CompAREdesign** implements Spearman’s correlation and Kendall’s tau as association measures. The former is much more frequent and is the one we recommend for time-to-event studies.

This package is restricted to two-arm RCTs. In future work, we consider expanding the R package to trials with more than two arms. In particular, we plan to extend the package to also include multi-arm trials where the efficacy of *K* treatments is tested against a shared control [22]. Additionally, the package could be expanded to include some adaptive features, such as sample size reassessment [7] and treatment selection [8]. Finally, the package could be enhanced to include trials with composite endpoints with more than two components.
